# Barriers to collaboration between health care, social services and schools

**DOI:** 10.5334/ijic.653

**Published:** 2011-09-16

**Authors:** Catharina Widmark, Christer Sandahl, Katarina Piuva, David Bergman

**Affiliations:** Karolinska Institutet, Medical Management Centre, SE-171 77 Stockholm, Sweden; Karolinska Institutet, Medical Management Centre, SE-171 77 Stockholm, Sweden; Department of Social Work, Stockholm University, SE-106 91 Stockholm, Sweden; Karolinska Institutet, Medical Management Centre, SE-171 77 Stockholm, Sweden

**Keywords:** obstacles to collaboration, perceptions, management, holding environment

## Abstract

**Background:**

It is essential for professionals from different organizations to collaborate when handling matters concerning children, adolescents, and their families in order to enable society to provide health care and social services from a comprehensive approach.

**Objective:**

This paper reports perceptions of obstacles to collaboration among professionals in health care (county council), social services (municipality), and schools in an administrative district of the city of Stockholm, Sweden.

**Methods:**

Data were collected in focus group interviews with unit managers and personnel.

**Results and discussion:**

Our results show that *allocation of responsibilities, confidence* and *the professional encounter* were areas where barriers to collaboration occurred, mainly depending on a *lack of clarity*. The responsibility for collaboration fell largely on the professionals and we found that *shared responsibility of managers from different organizations* is a crucial factor affecting successful collaboration. We conclude that a *holding environment*, as a social context that facilitates sense making, and a *committed management* would support these professionals in their efforts to collaborate.

## Introduction

### Collaboration in public service

Collaboration between different agencies in the welfare sector in matters related to mental health care of children and adolescents represents an area of considerable concern. In Sweden today this is particularly noticeable with respect to the increasing mental illness observed in this age group. First-line treatment is given to the approximately 20–30% of children and adolescents who need more support than is included in the general assistance provided by the community. Access to satisfactory health care and social services must be developed and that requires collaboration between professionals from different welfare sectors.

This study aimed at gaining a better understanding of what complicates collaboration between professionals from health care, social services and schools. We invited professionals to participate in focus group interviews to explore what perceptions they had on barriers to collaboration.

Previous research has highlighted organizational aspects of the obstacles to collaboration between professionals from different sectors of society, and according to Axelsson and colleagues [1, 2] these are both structural and cultural in nature.

The structural barriers include differences in the regulatory, financial, and administrative boundaries, and the cultural impediments consist of the various ways that the needs of individuals are considered, which are often a product of educational and organizational cultures. Axelsson and colleagues [1, 2] also pointed out that it is important for organizations to have a common interest, if they are to interact and have common goals. Freeth [3] found that collaboration is supported and maintained by shared responsibility for management, shared resources, and meetings between the stakeholders. Collective management responsibility entails leading the internal operations as well as the inter-organizational activities [4, 2], and it is a balancing act for collaborating managers to be loyal to both their own organization and the external counterparts. An altruistic approach [5] can be successful if the organization is considered in a wider context, that is, as an entity and in the longer term. Döös et al. [6] studied joint leadership among managers from the same or different units within an organization and observed that trust, shared values, and placing less emphasis on prestige are important prerequisites for successful collaborative leadership.

Axelsson and Axelsson [5] have described a number of ways of viewing the obstacles to collaboration between different professional groups. Such impediments can be due to mistrust caused by incomplete understanding between the parties concerned and to prejudice caused by territorial thinking. To achieve a more altruistic way of thinking, even among staff members, it is necessary to build trust between the individuals involved. These authors mean that attainment of that objective is the task of management. Having knowledge of one another is important for the ability to avoid unrealistic expectations and cross-border actions, which create barriers. Danermark and Kullberg [7] found a lack of equality between professional groups when they make different assessments on children’s needs from different models of explanations (psychological, medical, social and educational) that become a barrier. Common meeting places are an important prerequisite for exchanging information and experiences [8], and Willumsen [9] has also shown that leadership and collaboration are closely interlinked. An important task for managers is to motivate personnel to collaborate voluntarily and with full commitment. Ödegård and Strype [10] also found that motivation is one of the keys to personnels’ willingness to engage in collaboration. This is probably aided by providing clear information regarding joint goals and what is required of personnel to achieve successful collaboration [11, 12].

It has been argued that specialization in welfare services leads to fragmentation and the lack of a comprehensive view on people’s needs, and thus there is a risk that patients and clients will fall between the cracks [2, 13]. Specialized institutions in different public sectors need to interact and develop interdependent relationships [7]. Danermark et al. [8] have asserted that the demands for a comprehensive approach to human needs underline the importance of collaboration skills. This means that the parties involved require knowledge about barriers to cooperation in order to explain how to remove those obstacles or how to prevent them from occurring, and at the same time strengthen mechanisms that promote collaboration-promoting mechanisms. Danermark and colleagues [8] concluded that such knowledge is lacking at both the management and the personnel level, and that current inter-organizational collaboration is more like a trial and error process.

In summary, the research concerning collaboration between different welfare sectors of society have demonstrated the following:

- joint management of collaborating organizations is important [3];
- having a common interest is essential in collaboration [1, 2];
- the balancing act that managers must achieve and maintain collaboration [4, 2];
- it is vital that professionals are motivated for the joint task [9];
- trust should exist or be built between the professionals involved [5];
- managers and personnel should have a clear understanding of the factors that impede or promote collaboration [8].

Collaboration should be seen as a tool for achieving the objectives of the users, not as an end in itself for the professionals. Some researchers claim that the focus should be on the outcomes of the collaboration, not on the way the professionals interact with each other [14]. However, to gain a better understanding in this context, it is of interest to explore what complicates collaboration, since this process often is unsuccessful [15].

### Objectives

The aim of this study was to explore perceptions of barriers to collaboration between professionals working in different welfare sectors. More specifically, our objective was to address the following question:

- What perceptions do the professionals in health care, social services, and schools have on barriers to collaboration in the area of children and adolescent mental health?

## Methods

### Framework

We decided to conduct our research through focus group interviews with professionals from the welfare sector. This qualitative framework is a way to gain a deeper understanding of human thoughts and experiences. Interviewing in focus group is a valid method in that sense. Further, the method has the advantage of also having significance for the participants as they become conscious about and have the opportunity to discuss the subject of research, in this case difficulties of collaboration [16, 17].

### Data selection

This study was conducted in one of 14 administrative districts of the city of Stockholm, Sweden. It was considered as an appropriate and representative area for the study regarding mental health and the need for social and psychiatric support. The district had a population of approximately 48,000, and around 9500 of those inhabitants were children or adolescents aged 0–17 years. Statistics for 2009 indicated that the rate of sickness among adults (amount of days due to sickness) in this district was higher than in the city as a whole and was among the highest of all 14 districts. Moreover, the need for economic assistance in this district (5.1%) was somewhat greater compared to the city as a whole (4.0%) but was average compared to all the other districts. During the years 2007–2009, un-employment in the district increased from 2.9% to 4.8%, which was greater than the rise in the city as a whole. Around 460 children and adolescents had contact with the child and adolescent psychiatry (CAP) during the year.

We studied collaboration between professionals in health care (county council), social services (municipality), and schools (municipality) in the district of interest. The county council ran units responsible for the following: maternity health care, child health care, paediatric medical care, primary care, child habilitation and child and adolescent psychiatry. The social services dealt with matters related to children and adolescents with psychosocial problems, disabled children, leisure activities and child welfare. The district of interest had six primary schools and one secondary school under public administration. School is a place for all children and adolescents and as such is a significant party in collaboration. It is therefore studied as an individual party within the municipality. It was suggested that there had been a high degree of staff turnover in recent years at several of the public service units included in our study.

### Focus group interviews

Three persons, one from each of the studied organizations, were initially interviewed as a way of exploring relevant issues for discussion in the following focus group interviews.

To explore the perceptions of barriers to collaboration, we invited unit managers and personnel of all units in health care, social services, and schools to take part in focus group interviews to provide data for analysis. All unit managers were asked to participate themselves and to nominate one participant among their personnel, who was expected to have a minimum of two years of professional experience as well as experience of collaboration in the area. Six groups where created and conducted in November 2009. It was assumed that the participants would be more inclined to express their true opinions if they were assigned to groups including colleagues from their own area of work and organizational level rather than to mixed groups. Therefore, we divided the unit managers into three groups, each of which represented one of the three areas of public service examined in our study, and we divided the personnel into three groups in the same way (Table 1). Three questions were posed to the participants: “how are the county council/municipality/schools to interact with? What obstacles do you see in them? What obstacles are there in your own activity?”

All units within health care and social services, responsible for children aged 0–17 years, were represented in the focus groups. All primary schools and the secondary school were also represented. All participants signed an informed consent. The group discussions were conducted by the fourth author (DB) and the second author (CS) both employed at Karolinska Institutet. CS conducted the three groups with unit managers and DB the three with personnel. The research manager (first author, CW) was present as an observer during all six group discussions. This division of labour was a way to neutralize a possible bias as she (CW) also had experience as a former unit manager of the child and adolescent psychiatry in the district of interest.

### Analysis

Data from the six focus group interviews were audio-recorded, transcribed by a professional writing agency and then assessed by content analysis using an inductive approach. In an inductive approach in content analysis the researcher has an unbiased way of analysing the text. It is a way of describing differences and similarities in the text. One looks for manifest and latent content, or in other words, categories and themes. In this process the context has significance that makes knowledge important about the context in which the study is implemented [18, 19]. In this study this was enhanced by the researcher’s previous professional experience from the area.

Each transcript was read twice and then coded using Nvivo™, a computer program designed for coding text content. Seventy-seven codes were found, most of them similar for each focus group. Three categories emerged from data; *allocation of responsibilities*, *confidence* and *the professional encounter*. There were 39 codes in the first category, 17 in the second category and finally 21 codes in the third category. At several occasions the four authors discussed and compared codes and categories. All categories presented are the results of consensus decisions by the authors. A summary of the main findings were presented to the unit managers at an early stage of the analysis and we noted that they could well recognize the obstacles.

## Results

The main content of the data concerned the individual organization and communication between the organizations and between professionals. From an overall view our analysis showed, that collaboration worked well at times but poorly at others, and to a certain extent it lacked structure and common practices. The discussions in the focus groups revealed some experiences of successful collaboration, which were characterized by the following: professionals who listened to each other; common goals of collaboration on the cases of individual children; working in parallel with a division of labour based on a joint plan for support; listening to each other’s expertise through consultations; having good access to each other. The participants who were most critical of their inter-organizational collaboration used the words ‘catastrophe’ (personnel from the county council, PC) and ‘hornet’s nest’ (unit managers from the municipality, UMM) to describe it. The data also revealed a risk for clients and patients to fall between the cracks that were consistent with previous research in the area.

The first of the categories that emerged from the content analysis comprised *allocation of responsibilities* between the three public service areas in relation to children and adolescents with mental illness. Ambiguity in this regard appeared to have the greatest negative impact on collaboration. Various structural conditions were identified as barriers to collaboration. The second category was *confidence*; briefly, lack of knowledge of other activities affected trust between professionals and became an obstacle. The third category concerned *the professional encounter*; more precisely, structural differences between the three service areas affected communication between respective professionals and were perceived as impediments.

The perceptions of barriers to collaboration were mainly similar among the unit managers and the personnel regarding the three categories mentioned above. The personnel mentioned the economy and the unit managers also mentioned political decisions as factors that have an impact on collaboration, and this was expressed in isolated comments. Therefore, these results are presented here in a more coherent form.

### Allocation of responsibilities

The organizations we studied had widely varying missions and regulatory framework and offered different kinds of services. Some of the activities target all children (general services, i.e. schools, child health care), whereas others applied to specific groups (specialist services, i.e. paedriatic medical care, child and adolescent psychiatry) and could be mandatory (schools) or voluntary (activities within health care and social services, which also had the authority to make decisions about children and adolescents without parental consent). Obscure and unspoken differences between the organizations were perceived as a grey area, where the allocation of responsibilities became unclear. The meaning of each other’s mission appeared to be unclear and required clarification to be fully understood. Furthermore, there seemed to be ambivalence about confidentiality rules, which were handled differently by the different organizations. The lack of clarity became an obstacle in the interaction.

*“It’s the parents’ problem and the social services’. But if the child actually has a diagnosis, then there’s probably no one who knows whose problem it is.”* (personnel from the school, PS).

The obligation for all professionals in the area of child and adolescent care to inform the municipality about their concerns regarding a particular child did not always give the desired feedback. Indeed, it could create uncertainty about whether or not the information was perceived as adequate, and if it had led to needed support. On the other hand, there was an understanding among the municipal professionals that the lack of feedback affected confidence, although this was not completely established.

*“Then you know, after you’ve filed the complaint, you can wonder ‘what happened after that, didn’t they think this was as serious as we did?’”* (PS)

Specialist activities within health care were aimed at groups or individuals with specific needs, and the accessibility for children and adolescents therefore varied between this kind of activity and general activities. In other words; the threshold levels varied. This also applied to professionals who referred clients or patients to specialist activities. The study participants regarded this as an ambiguity and felt that it caused the professionals to have prejudiced and oversimplified perceptions of each other.

*”There is not anyone who can get [access] to CAP [child and adolescent psychiatry], but they should fit into…* (PS)

*”Yes, they choose a bit”.* (PS)

The professionals had to delimit their efforts for children and adolescents due to declining resources. This aspect seemed to influence interactions between the professionals, but there was also an understanding of it. The range of support was shrinking along with imposed decreases in resources. Furthermore, this resulted in inequality between professional groups, when the view of one group with respect to a child’s need for support was not backed up by another group due to the lack of resources. There was no room for the professionals to argue with each other about what was best for the child. The solution was given.

*“… we used to be able to make demands or fight with the school in a more equal way, but now I think it’s pretty clear that they’ve been given very limited resources.”* (unit managers from the municipality, UMM)

To summarize, there were obvious *ambiguities* in the differences in responsibilities between the organizations and the issues related to differences in missions and regulations. The professionals became *uncertain* of what they could expect from the other organizations. It also seemed that this contributed to a tendency for the professionals to develop biased and superficial perceptions of each other’s activities.

### Confidence

Trust between the interacting unit managers and personnel across the units were apparently insecure. This was exemplified by the uncertainty that was expressed by some participants regarding the facts on which other professionals based their assessment of a child’s situation. In addition, some described being dissatisfied because their own assessments were not taken seriously by professionals from other organizations.

*“… they describe the situation, but they themselves haven’t seen that there are special needs / … / to my way of thinking, that indicates a lack of knowledge.”* (personnel from the municipality, PM)

Professionals sometimes felt that their collaboration partner lacked commitment. This was experienced as one party’s lack of interest (i.e. social service) in what support the other partner (i.e. school) could provide or in what knowledge there was concerning a particular child. On the other hand, there were mutual expectations among professionals about how other agencies could assist a child, and these could be unrealistic or based on negative experiences. In the worst case, expectations were said to involve cross-border actions in which one professional commented on what the other professional ought to do.

*“They think we should do things that we can’t always do. The expectations for the school are kind of unrealistic.”* (PS)

The discussions indicated that professionals (i.e. from the social services) who were overworked sometimes verbalized their frustrations when talking with a collaboration partner (i.e. from the school). That situation was very well understood by the other partner, who also felt a concern about what support a child could get from an overworked partner.

*“‘Yeah, I’m totally overloaded, and I’m going to resign soon,’ and you’re not all that interested in hearing that when you have to collaborate on a child’s case, so you almost have to sit there and console those poor people who are completely worn out.”* (UMS)

In summary, there were questions about confidence between the professionals in the organizations included in this study. This situation was also affected by a certain lack of knowledge about the other professionals’ skills in assessing children’s needs, about their way of working and about the resources the other organizations could provide for a child. If one party in an interaction was perceived as uncommitted, the uncertainty of the interplay was reinforced.

### The professional encounter

Different organizations use different models (psychological, medical, social and educational) to explain how work with children and young people should be conducted. The organizations with disparate missions require different skills and practices of the professionals. Children’s needs are viewed from different perspectives, and these dissimilarities have to be made visible. However, this was apparently not very noticeable among those collaborating in the organizations we investigated. The professionals talked at cross purposes due to lack of clarity, and thus the necessary consensus failed.

*“Social Services may think that we see too many needs at CAP [child and adolescent psychiatry], you know, psychological needs, but that are very important, but aren’t within the responsibility of the social services /…/you /…/ see some other needs, which is not so clear on one or two meetings. There I believe that a collision can be between the Social Services and CAP.”* (personnel from the county council, PC).

The common practices in collaboration seemed to be unclear. When the aim of a joint meeting was not specified, people with diverse decision mandates were called to take part, and their expectations varied as to what the meeting would lead to. This was particularly apparent concerning economic issues. Also, if a meeting did not produce the expected solutions, in some cases it was necessary to make new attempts to reach agreement. Collaboration was circumstantial and time consuming.

*“… we’re there as professional managers, but those representing the other side are not professional managers, and that’s an obstacle to efficiency.”* (UMS)

The accessibility to different organizations varied, but, despite that, the accessibility of professionals to each other must be as smooth as possible. However, in the present context there seemed to be a tendency for some people to ignore the criteria of other organizations, which probably led to unnecessary diversions. Furthermore, when the professionals did not acknowledge each other’s attempts to make contact, it was perceived as a repudiation, and the same applied to when professionals in one organization guarded their territory and were reluctant to collaborate.

*“… they’d prefer that we didn’t interfere / … / yeah, they’re kind of nonchalant.”* (PC)

For some time, there had been high staff turnover or downsizing in different parts of the studied organizations. All of the participants felt that this had led to a break in continuity of contact between professionals from different organizations. Effective personal relationships across organizational boundaries had been disrupted, and new ones had to be built. This situation had led to a loss of knowledge between the professionals and contributed to uncertainty in collaboration. It had also contributed to making collaboration too detailed and time consuming.

*“There are many new [personnel] that have to be trained and don’t know, and that means there is lack of knowledge…”* (UMS)

In summary, the *lack of clarity* about the differences in both culture and structure between the principal stakeholders affected the professionals’ interactions. Collaboration was too comprehensive and time consuming, and those who were involved tended to be hesitant about or opposed to communication.

Three organizational levels are mentioned in this context. Firstly personnel and secondly unit managers, who participated in the focus group interviews and discussed their perceptions of barriers to collaboration. Our analysis showed that unit managers were equally involved in collaboration as their personnel. In this context ‘managers’ are described as ߢtop managers’ on the third top level in each organization.

### Summary of results

**Figure 1:**
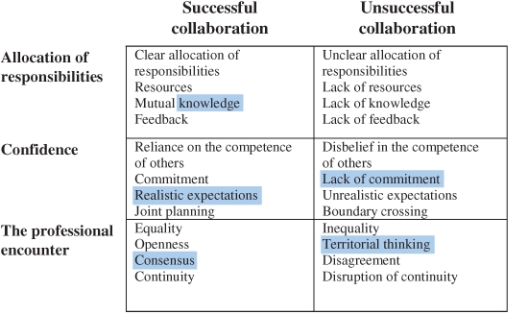
A tentative model for successful and unsuccessful collaboration.

The impression was that the responsibility for collaboration rested on the shoulders of the professionals (i.e. unit managers and personnel). They appeared to be expected to handle the task of collaboration on their own, and had poor support in this regard from their top managers. In general, most participants did not mention the top management at all. It is noticeable since the literature point to the importance of support from the management for successful collaboration. We summarize our results in a tentative model for successful and unsuccessful collaboration (Figure 1).

## Discussion

The aim of this study was to explore public service professionals’ perceptions of barriers to collaboration in matters concerning children and adolescents. We summarized the experiences and impressions they shared in three categories: *allocation of responsibilities*, *confidence* and *the professional encounter*.

The first category (allocation of responsibilities) shows that the participants perceived the ambiguity of the differences in mandates and regulations as an obstacle, and these differences seemed to be implicit and create uncertainty in interactions. The second category (confidence) demonstrates that trust between collaborating stakeholders was affected by lack of knowledge of the skills and resources and spheres of action of other professional groups. When one collaborating party lacked commitment for a child, it affected confidence and led to indecisiveness in the joint work. The third category (the professional encounter) indicates that unspoken differences in the approach used to address children’s needs (explanatory models) in some cases led to misunderstandings between the professionals involved. Communication was also affected by dissimilarities in the missions and criteria (thresholds) of the stakeholders, and those differences were perceived as barriers that created frustration.

A recurring theme in the focus groups was the *ambiguity* that existed regarding the differences between the main stakeholders and how they collaborate with each other. Boklund [20] has concluded that there is a *demand for clarity* between cooperating professionals, and it appears that this aspect may have *created uncertainty* in the collaborations in the present context. Furthermore, according to Axelsson and Axelsson [5], such uncertainty is associated with *knowledge* of other activities and ways of working, and it plays an important role in the *trust* between the parties involved. An aspect that should be considered is to what extent this knowledge is or is not declared (articulated) among those who are participating in collaboration [21]*. Openly expressing knowledge* of other parties is likely to bring coherence in the collaboration and the goals of the joint work, and also concerning what is required of the professionals [11, 12]. To some extent the professionals in the current focus groups lacked such knowledge about each other. They tended to guard their activity limits and have unrealistic expectations, and even seemed to have adopted shallow and prejudiced perceptions about other parties. These deficiencies affected communication among the professionals and led to misunderstandings in discussions about children’s needs.

Despite this diversity of obstructive factors, the participating professionals indicated that they also experienced successful collaboration. They perceived themselves as being sympathetic towards working together with other professionals, and they even considered that to be important in matters involving children and young people.

How can we explain this contradiction? What prevented these public service professionals from removing barriers and developing successful strategies to maintain long-term collaboration? According to Willumsen [9], collaboration and management are closely interlinked. The extent to which ambiguity and lack of knowledge exists can no doubt be linked to the management responsibilities.

It is known that shared responsibility of managers from different organizations is a crucial factor affecting the way that collaboration is implemented and maintained. The *commitment* of such professionals is required to initiate collaboration, define common goals, achieve follow-up, and motivate personnel [1–3, 9–12]. Furthermore, it has been emphasized that the demand for *collaboration skills* is a prerequisite for successful collaboration [8].

Committed and skilled managers working together in collaboration can be seen as creating a ‘holding environment’ that represents a workspace [22] in which professionals can develop collaboration. A holding environment is defined as “a social context that reduces disturbing affect and facilitates sense making” [22, page 50] and has influence on interpersonal processes, social structures and cultures. This kind of environment appeared to be absent in the present context, which might also be interpreted as a reflection of the professionals’ reality. If that was indeed the case, then there was no support from management, or the assistance that was provided was too imprecise and probably also too implicit to allow creation of successful, sustainable and long-term collaboration.

Based on the arguments above, it can be assumed that committed managers may provide a holding environment in which collaboration is implemented and monitored in a successful way. It is expected that *clarity* [20] can be achieved in joint work by explaining the conditions so that the professionals can understand the assigned tasks and goals [10]. This means that creating a holding environment and clarifying the conditions for collaboration will make it possible to establish *confidence* between professionals from different organizations and will also enable intercommunication based on equality and openness. Uncommitted management is likely to lead to a disengaged environment and ambiguity in collaboration. Unspoken conditions may produce uncertainty between professionals and lead to manifest territorial thinking.

### Limitations

A case study of this kind cannot give a comprehensive and general answer to the research question posed. A number of limitations could be put forward, for example the fact that the research manager (first author CW) was a former unit manager of one of the studied organizations. Her previous experiences might have had impact on the focus group interviews, which was balanced by two of the other authors (CS and DB) conducted the interviews and the research manager acted as an observer. It might also have influenced her conclusions and interpretations of the data. Depth interviews could have been another way of collecting data to gain information that was hidden in a group discussion. Differences between organizations were after all even a difference between the groups and might be another limitation. Groups from the municipality were more homogeneous than groups from the county council regarding geographical location and educational background. Groups from school were also more homogeneous despite the geographical spread of schools.

## Conclusions

This study revealed professionals’ perceptions of barriers to collaboration and the findings agree with previous reports on the subject, primarily Swedish but also comparable studies in other countries. A keyword for the professionals was *clarity*, considering knowledge of other parties regarding aspects such as differences in mandates and regulations, allocation of responsibilities, competence, explanatory models and working approaches.

It is suggested that collaboration between professionals from different organizations requires *committed management* in order to be successful. Moreover, collaboration requires *shared* management that includes *collaboration skills* to promote declaration of the goals of the shared efforts, which could as well help define what is required of the professionals involved. A *holding environment*, with clear objectives to the long-term support of collaboration, would eliminate many of the barriers that participants in the study expressed. The result also points to an interesting perspective regarding users’ views on professionals interacting in collaboration in child and adolescent mental health care.

## Figures and Tables

**Table 1.** Composition of the six focus groups

Unit managers from the county council (UMC)	Personnel from the county council (PC)
(n=6 persons; 5 women, 1 man)	(n=6 persons; all women)
Unit managers from the municipality (UMM)	Personnel from the municipality (PM)
(n=5 persons; 3 women, 2 men)	(n=4 persons; 3 women, 1 man)
Unit managers from the school (UMS)	Personnel from the school (PS)
(n=6 person; 3 women, 3 men)	(n=5 persons; all women)
Unit managers total n=17 (11 women, 6 men)	Personnel total n=15 persons (14 women, 1 man)
